# Deep learning for terahertz image denoising in nondestructive historical document analysis

**DOI:** 10.1038/s41598-022-26957-7

**Published:** 2022-12-29

**Authors:** Balaka Dutta, Konstantin Root, Ingrid Ullmann, Fabian Wagner, Martin Mayr, Mathias Seuret, Mareike Thies, Daniel Stromer, Vincent Christlein, Jan Schür, Andreas Maier, Yixing Huang

**Affiliations:** 1 Pattern Recognition Lab, Friedrich-Alexander-Universität Erlangen-Nürnberg, Erlangen, Germany; 2 Institute of Microwaves and Photonics, Friedrich-Alexander-Universität Erlangen-Nürnberg, Erlangen, Germany; 3 Siemens Healthcare GmbH, Erlangen, Germany; 4 Department of Radiation Oncology, University Hospital Erlangen, Friedrich-Alexander-Universität Erlangen-Nürnberg, Erlangen, Germany

**Keywords:** Computer science, Imaging techniques

## Abstract

Historical documents contain essential information about the past, including places, people, or events. Many of these valuable cultural artifacts cannot be further examined due to aging or external influences, as they are too fragile to be opened or turned over, so their rich contents remain hidden. Terahertz (THz) imaging is a nondestructive 3D imaging technique that can be used to reveal the hidden contents without damaging the documents. As noise or imaging artifacts are predominantly present in reconstructed images processed by standard THz reconstruction algorithms, this work intends to improve THz image quality with deep learning. To overcome the data scarcity problem in training a supervised deep learning model, an unsupervised deep learning network (CycleGAN) is first applied to generate paired noisy THz images from clean images (clean images are generated by a handwriting generator). With such synthetic noisy-to-clean paired images, a supervised deep learning model using Pix2pixGAN is trained, which is effective to enhance real noisy THz images. After Pix2pixGAN denoising, 99% characters written on one-side of the Xuan paper can be clearly recognized, while 61% characters written on one-side of the standard paper are sufficiently recognized. The average perceptual indices of Pix2pixGAN processed images are 16.83, which is very close to the average perceptual index 16.19 of clean handwriting images. Our work has important value for THz-imaging-based nondestructive historical document analysis.

## Introduction

Historical documents are the original records containing valuable information about an event, object, person, or work of art from the past, which can serve as primary sources for important ingredients of the historical methodology^1^. They help us to understand people and societies and the changes they have undergone, reflecting on the ways people built their lives in the past. Studying their stories helps us to develop a moral understanding of how to live our own lives in the present. They provide information on our past mistakes, allowing us to create a better future.

Historical documents are found in various formats, such as letters, diaries, newspapers, and journals, to name a few. Due to aging and storing conditions, these documents are very fragile and hence challenging to retrieve valuable information in a regular way. For example, an old letter paper contained in an old envelope will be easily damaged or fully destroyed, if it is read in a conventional way after opening the envelope. Therefore, non-invasive imaging for historical document analysis is highly desired^2,3^. Such imaging techniques range from X-rays (wavelength: 0.01 nm to 10 nm) to Terahertz (THz) waves (wavelength: 100 µm to 1 mm)^4^ on the energy spectrum. 3D X-ray computed tomography (CT)^3,5^ is an effective approach to digitizing historical documents because of its high transmission ability and high imaging resolution. However, it has the risk of accelerating the aging process due to the X-ray ionizing radiation during the scanning procedure. Imaging techniques using ultraviolet light to infrared lights have no radiation risk^1,2,6^, but their low transmission ability prohibits them from retrieving information from concealed documents. Nowadays THz waves have been widely applied to various fields^7–9^ such as wireless communications^10^, security check^11^, medical imaging^12^ and nondestructive testing^13^. THz imaging allows the measurement of the thickness, density, and structural properties of various materials. Its high transmission property and non-ionization exposure make it an emerging technique for contact-free, noninvasive imaging in historical document analysis^14–16^. Because of the relatively long wavelength, THz imaging typically has the image resolution of a few hundred microns^17^, which is much lower than X-ray and optical imaging, but is still sufficient for historical document analysis. Although THz imaging is challenging for scanning thick books, it is promising to extract information from documents consisting of a few paper layers like letters and papyrus scrolls^15^.

Despite its clear advantages, THz imaging requires a trade-off between image quality and imaging speed^18^. THz images typically suffer from speckle noise^19^, especially in a fast imaging mode. Therefore, THz image denoising has an important value in practical applications. Various conventional algorithms have been applied to THz image enhancement such as adaptive filtering^20–22^ and deconvolution methods^23–25^. Adaptive filtering filters out high-frequency noise while preserving the sharpness of edges. Deconvolution methods enhance THz image resolution and suppress noise based on the accurate modelling of the point spread function^23^. Compressed sensing techniques have also been widely investigated in THz image reconstruction^18,26–29^. As compressed sensing is able to reconstruct images from relatively few measurements by the exploitation of sparsity, it has been demonstrated effective for high-speed THz imaging, like single-pixel THz imaging systems^28,29^. For example, Li et al.^18^ proposed to combine the ant colony algorithm with a compressive sensing technique based on local Fourier transform, which reduces noise well while preserving edge information.

Recently, deep learning has achieved impressive results in various fields, including THz imaging^30^. Deep learning has been applied to segmentation and classification tasks in THz images such as impurity detection in wheat^31,32^, breast cancer classification^33^, and heavy-metal detection in soils^34^. The low resolution problem of THz imaging can also be mitigated by deep learning based super-resolution techniques^35,36^. In rapid THz imaging, deep learning can significantly reduce algorithm complexity and increase signal-to-noise ratio^37–42^. For example, Ljubenović et al.^37^ used a convolutional neural network (CNN) for THz image deblurring and their work demonstrates the efficacy of CNNs for denoising on synthetic THz data. Choi et al.^42^ adopted the WaveNet from the field of speech and audio for THz image denoising in the frequency domain for 1D temporal signals. To overcome limited training data, Jiao et al.^43^ proposed a Noise2Noise-based network for THz spectrum denoising using transfer learning from low-quality underwater images. However, deep learning has not been investigated in THz imaging for historical document analysis yet.

The paper aims to improve THz image quality for historical document analysis by reducing imaging noise and artifacts, which commonly exist in reconstructed images processed by standard THz reconstruction algorithms. Our work demonstrates the feasibility of THz imaging in information retrieval from sealed envelopes. It also demonstrates the efficacy of deep learning for THz image enhancement for better character recognition. To the best of our knowledge, our work is the first to apply deep learning to THz image enhancement for historical document analysis. Our experiments indicate that the deep learning enhanced image quality relies on the paper type and the page sides, which is valuable information conveyed to the community. From our point of view, our work is a very important step towards real applications of THz imaging in nondestructive document analysis, which will encourage more research in this topic.

## Materials

The THz images used in this work were acquired at the Institute of Microwaves and Photonics (LHFT), Friedrich-Alexander-Universität Erlangen-Nürnberg, Erlangen, Germany. For the measurements, the commercial radar imaging system “Quality Automotive Radome Tester” by Rohde and Schwarz was used. It is a multiple-input-multiple-output (MIMO) radar consisting of $3\times 4$ sparse subarrays with 1128 transmit channels and 1128 receive channels in total. The applied signal is a 64-point stepped-frequency continuous-wave signal, in the range of 74 GHz to 79 GHz. More details of the scanner can be found here (https://www.rohde-schwarz.com/us/product/qar).

**Figure 1:**
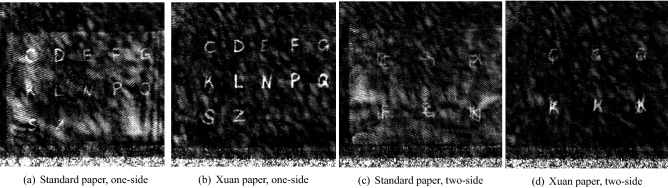
Examples of THz images from one-side-standard paper (**a**), one-side-Xuan paper (**b**), two-side-standard paper (**c**) and two-side-Xuan paper (**d**).

To mimic historic letters concealed in envelopes, two types of paper are used to create the images for the dataset. One dataset was made with A4 standard paper and the other with the A4 Xuan-Paper. The Xuan-Paper features great tensile strength, smooth surface, pure and clean texture, clean stroke, and excellent resistance to corrosion, moth, and mold. The Xuan-paper is thinner than the standard paper and hence the corresponding Xuan-paper THz images have less noise than the standard-paper THz images. In addition, the papers were written in two ways: one was written on a single side and the other was on both sides. Therefore the two-side written images contain overlapping letters. All the letters were written with the calligraphy ink Type 29770 from Rohrer & Klinger Company. For each letter, a 3-D volume was reconstructed with a size of $705 \times 1025 \times 97$ voxels and an anisotropic voxel spacing of $0.5\times 0.5\times 0.573\,\text {mm}^3$. To reduce the effect of paper wrinkles and tilt, maximum intensity projection along the Z-direction was used to convert 3-D volumes to 2-D images. Two THz image examples from the standard paper and the Xuan paper are displayed in Fig. 1a,b, respectively. The THz signal is emitted and caught by a Vector Network Analyzer(VNA) (Rohde & Schwarz ZVA 24) combined with frequency extenders (Rohde & Schwarz ZVA-Z325) for the range between 220 and 325 GHz^15^. Two spline horn antennas and two polyethylene dielectric lenses were also used to achieve optimal focusing.

## Methods

As displayed in Fig. 1, the acquired THz images suffer from severe noise, which is an obstacle to the recognition of context in historic document analysis. This work aims to enhance THz images using deep learning methods. Since THz image acquisition is expensive and time-consuming, it is challenging to acquire paired clean and noisy THz images to train a supervised deep learning model. To overcome the lack of paired data, we propose to apply an unsupervised learning network, in particular CycleGAN, to generate paired images using unpaired synthetic clean images and real noisy images. The synthetic clean images are generated by a handwriting generator, and a learned CycleGAN model will add similar noise patterns into the synthetic clean images to construct clean and noisy image pairs. With such paired images, a supervised learning network, in particular Pix2pixGAN, is applied for final THz image denoising.

### Handwriting data generation

**Figure 2:**
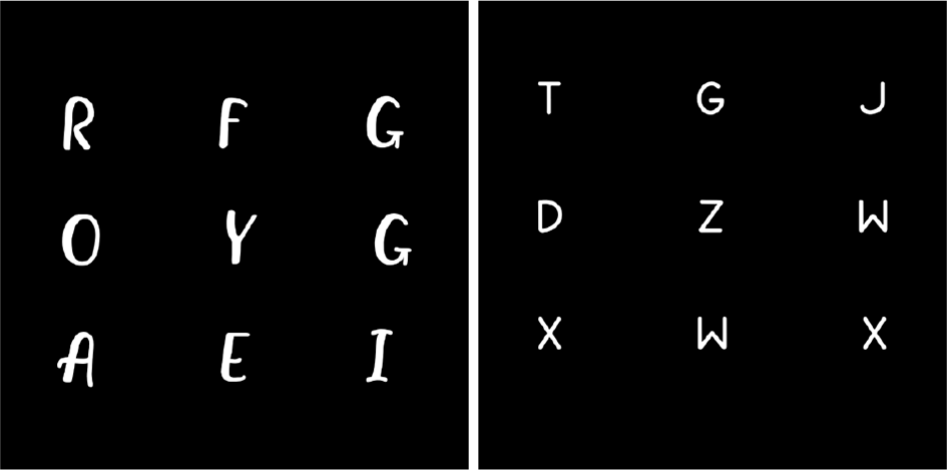
Two exemplary images generated by the handwriting generator with two different fonts.

Supervised deep learning-based algorithms require paired data for the model to learn the relationship between clean and noisy images. Our dataset consists of noisy THz images only. Hence, a handwriting generator^44^ was employed to generate clean handwriting images. A black background was taken, and random letters in white were created over it using random fonts. 2000 clean images in total are created to train our models as the first step result. The outputs of the handwriting generator are binary images of letters with different font types. They are saved in 8-bit PNG format. Figure 2 displays two exemplary images generated by the handwriting generator with two different fonts.

### Synthesis of paired data via CycleGAN using unpaired data

Conversion between clean and noisy images is fundamentally an image-to-image translation task. Since only unpaired instead of paired synthetic clean images and real noisy THz images are available, CycleGAN^45^ is applied for such unpaired image-to-image translation in this work. CycleGAN consists of two generators, $G_{AB}$ that transfers an image from domain *A* to *B* and $G_{BA}$ that transfers an image from domain *B* to *A*. In particular in our work, domain *A* contains clean text images generated by the handwriting generator and domain *B* contains images with THz imaging noise and artifacts. Two discriminators $D_A$ and $D_B$ distinguish whether an image belongs to that domain. For a pair of $G_{AB}$ and $D_B$, the adversarial loss function is defined as,

1 $$\begin{aligned} \begin{array}{l} \mathscr {L}_{\text {GAN}}(G_{AB}, D_B) = \mathbb {E}_{b\sim p_{B(b)}}[\log D_B(b)] + \mathbb {E}_{a\sim p_{A(a)}}[1-\log D_B(G_{AB}(a))]. \end{array} \end{aligned}$$

Similarly the adversarial loss for $G_{BA}$ and $D_A$ is defined as $\mathscr {L}_{\text {GAN}}(G_{BA}, D_A)$. In addition, a cycle-consistency loss is applied to minimize the reconstruction error after an image of one domain to another is translated back to the original domain,

2 $$\begin{aligned} \begin{array}{l} \mathscr {L}_{\text {cyc}}(G_{AB}, G_{BA}) = \mathbb {E}_{a\sim p_{A(a)}}[||a - G_{BA}(G_{AB}(a))||_1] + \mathbb {E}_{b\sim p_{B(b)}}[||b - G_{AB}(G_{BA}(b))||_1]. \end{array} \end{aligned}$$

The overall objective function is,

3 $$\begin{aligned} \begin{array}{l} G_{AB}^*, G_{BA}^*= \arg \min _{G_{AB}, G_{BA}} \max _{D_A, D_B} \mathscr {L}_{\text {GAN}}(G_{AB}, D_B) + \mathscr {L}_{\text {GAN}}(G_{BA}, D_A) + \lambda _\text {cyc} \mathscr {L}_{\text {cyc}}(G_{AB}, G_{BA}). \end{array} \end{aligned}$$

**Figure 3:**
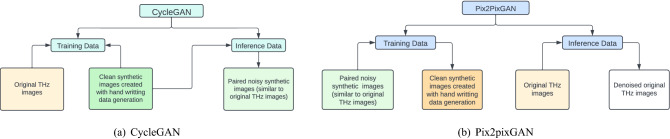
Proposed pipelines for synthetic data generation using CycleGAN (**a**) and for THz denoising using Pix2PixGAN (**b**).

In our work, during training we kept clean synthetic images created via our handwriting generator in one domain and kept the collected real THz images in the other domain, as displayed in Fig. 3a. During inference, the clean synthetic images are reused as the input test data, and CycleGAN outputs their corresponding paired noisy images, which share similar noise characteristics to the real noisy THz images.

Note that during inference, the real noisy THz images can be used as the input data as well and CycleGAN will output their corresponding denoised images. In this work, such direct denoising by CycleGAN is also investigated.

### Image denoising using Pix2PixGAN

In this work, Pix2pixGAN^46^ is applied to translate noisy THz images to denoised ones with paired data. Pix2pixGAN is a conditional GAN, which uses the U-Net as the generator, *G* and a 5-layer patch-wise convolutional classifier as the discriminator, *D*. *G* learns to convert noisy THz images into clean ones. *D* learns to distinguish the output denoised images from reference clean images. The objective of the conditional GAN is,

4 $$\begin{aligned} \begin{array}{l} \mathscr {L}_{\text {cGAN}}(G,D) = \mathbb {E}_{\varvec{x},\varvec{y}}\left[ \log {D(\varvec{x}, \varvec{y})}\right] + \mathbb {E}_{\varvec{x}}\left[ \log {\left( 1 - D(\varvec{x}, G(\varvec{x})\right) }\right] , \end{array} \end{aligned}$$

where $\varvec{x}$ is the input, $\varvec{y}$ is the target, *G* tries to minimize this objective against an adversarial *D* that tries to maximize it, i.e., $G^{*}= \arg \min _{G}\max _{D}\mathscr {L}_{\text {cGAN}}(G,D)$. In addition, an $\ell _1$ loss function is applied to train the generator’s output close to the target with less blurring compared to $\ell _2$ loss,

5 $$\begin{aligned} \mathscr {L}_{\ell _1}=\mathbb {E}_{\varvec{x},\varvec{y}}\left[ ||\varvec{y}- G(\varvec{x})||_1 \right] . \end{aligned}$$

The overall objective function is

6 $$\begin{aligned} G^*= \arg \min _G \max _D \mathscr {L}_{\text {cGAN}}(G,D) + \lambda _1 \mathscr {L}_{\ell _1}. \end{aligned}$$

As displayed in Fig. 3b, during training the synthetic noisy images from CycleGAN are used as the input and the corresponding clean images from the handwriting generator are used as the target. Only synthetic images are used for training. During inference, the real noisy THz images are used as the input and Pix2pixGAN predicts their corresponding denoised versions.

## Experimental setup

### Training data synthesis using CycleGAN

The synthetic dataset was created using CycleGAN. For this experiment, the code from Jun-Yan Zhu et al.^45^ available on GitHub was adopted. The basic model for the discriminator is a PatchGAN, with a patch of size $70 \times 70$ and a 9-layer ResNet as the generator. The dataset consisted of two domains, clean synthetic images created by the handwriting data generator and the original THz images. The model was trained using an Adam optimizer with a batch size of 2 for 200 epochs with an initial learning rate of 0.0002 to generate 2000 noisy synthetic images similar to the initial THz images. The weight for the cycle-consistent loss $\lambda _\text {cyc}$ is set to 0.5. For the generator, no dropout was applied. The input channel and output channel were both set to 1. The learning rate was kept the same for the first 100 epochs and linearly decayed to zero over the following 100 epochs. All the images were resized and cropped to $256 \times 256$ during data preprocessing, and no data augmentation was used. The rest of the parameters were kept unchanged with respect to^45^.

### Image denoising using Pix2pixGAN

**Figure 4:**
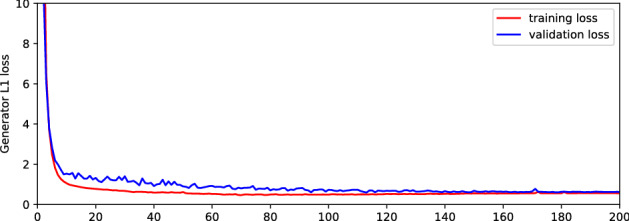
The plot of training and validation losses for training Pix2pixGAN.

The U-Net is used as the Pix2pixGAN generator, which contains 8 down-sampling modules as well as 8 skip connections. For more details, please refer to the “unet-256” configuration in the authors’ implementation^46^. An Adam optimizer was used to train the model with a batch size of 5 for 200 epochs with a constant learning rate of 0.0002. The weight for the $\ell _1$ loss was set to 100. It was trained with the 2000 paired noisy synthetic THz images created using CycleGAN, and the inference dataset consisted of the 34 original THz images. A validation dataset of 30 paired noisy synthetic THz images is used to monitor overfitting. The training and validation $\ell _1$ losses of the generator are displayed in Fig. 4, where no obvious overfitting occurs. As proposed in the paper^46^, random jitter was applied by resizing the $256 \times 256$ input images to $286 \times 286$ and then randomly cropping them back to size $256 \times 256$. The model weights were initialized following a Gaussian distribution with zero mean and standard deviation 0.02. The remaining parameters were kept the same as the standard version^46^.

### Comparison algorithms

In this work, some exemplary results of other algorithms are also displayed as a comparison. The bilateral filter^47^ and its trainable version^48^ are applied to compare with well-known adaptive filters. In particular trainable bilateral filter versions have been shown to provide robust denoising performance in the context of medical imaging^49^. The iterative reweighted total variation (wTV) algorithm^50^ is selected as a compressed sensing representative. The half instance normalization network (HINet)^51^ is chosen as a general deep learning denoising representative. Self-supervised learning algorithms do not rely on labelled training data, which can avoid the data scarcity problem. In this work, three self-supervised learning algorithms are selected: Noise2Self^52^, Noise2Void and Self-supervised vision transformer (SiT)^53^. SiT applies the latest techniques of transformers. Noise2Self and Noise2Void are well-known self-supervised denoising algorithms. In our experiments, three trainable bilateral filter layers are trained in a self-supervised way using the Noise2Void method following the setup of Wagner et al.^48^.

### Evaluation metrics

Since ground truth images are not available for the CycleGAN synthetic images and the denoised real THz images, a non-reference image quality metric called perceptual index (PI)^54^ is used to quantify these images. The perceptual index is calculated from the non-reference metrics of the natural image quality evaluator (NIQE)^55^ and the Ma’s score^56^,

7 $$\begin{aligned} \text {PI} = \frac{1}{2}\left( (10 - \text {Ma}) + \text {NIQE} \right) , \end{aligned}$$

both of which extract image features to compute the perceptual quality. For super resolution tasks on natural images, a lower PI value corresponds to richer fine structures and hence indicates better perceptual quality. In our application, a lower PI value corresponds to more high-frequency noise/artifacts in general. The average PI value of all the original noisy THz images is 6.85 with a standard deviation of 0.60, while that of the clean handwriting generator images is 16.19 with a standard deviation of 0.45. Therefore, larger PI scores are desired for our denoising results.

In addition, a custom approach is applied to quantify the algorithms used to denoise the THz images. As this paper aims to reduce the noise of THz images and finally retrieve the original data ideally or at least its structure, the characters visible with bare eyes are counted as a success, and if a character, any part of it or the entire character was missing, it is not considered as a valid output. The same accuracy calculation has been followed in the case of overlapping characters. Two overlapped characters count as a single structure for both-sided written images, so it is impossible to identify the characters separately in this case. The correct retrieval of overlapped characters’ structure is counted as a success. The results are differentiated by the type of paper.

8 $$\begin{aligned} \text{ Accuracy } =\frac{\text{ Number } \text{ of } \text{ character(s) } \text{ retrieved } }{ \text{ Number } \text{ of } \text{ character(s) } \text{ present } \text{ in } \text{ image } } \times 100 \end{aligned}$$

The accuracy is measured according to Eq. (8), and a comparative result is displayed in Table 2 for the Xuan-Paper and standard paper.

## Results

### CycleGAN results

**Figure 5:**
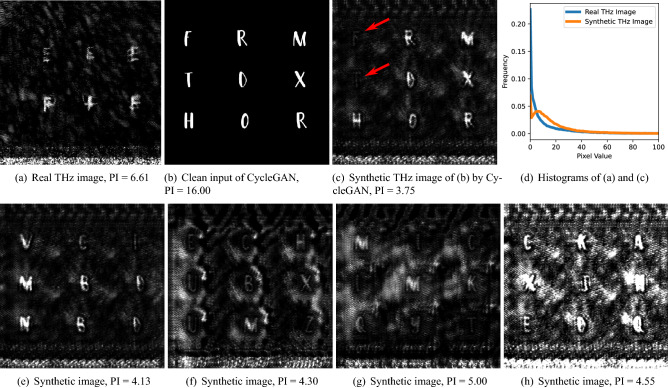
An exemplary real THz image and five exemplary synthetic THz images generated by CycleGAN along with the clean synthetic image. The characters indicated by the arrows in (**c**) are not fully visible. The perceptual index (PI) for each synthetic image is displayed in the corresponding subcaption.

**Figure 6:**
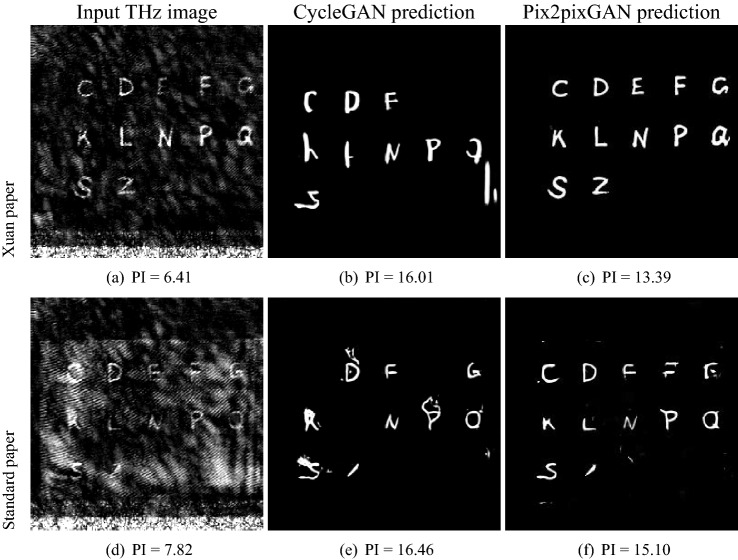
The CycleGAN and Pix2pixGAN results of two exemplary real THz images written on Xuan paper (top row) and standard paper (bottom row), respectively.

One exemplary synthetic image from CycleGAN is displayed in Fig. 5c together with its corresponding clean input image Fig. 5b and a real THz image Fig. 5a. Figure 5a,c have similar appearance, although the two characters indicated by the arrows are hardly visible. The histograms of Fig. 5a,c are displayed in Fig. 5d, which indicates that the synthetic image also has similar intensity distributions to the real THz image. The average mean intensity, average standard deviation, and average total variation (TV) values for all the real and synthetic images are displayed in Table 1. For all the synthetic images, the average perceptual index is 4.52 with a standard deviation of 0.83. To show the overall appearances of the synthetic images, four additional synthetic images together with their PI values are displayed in Fig. 6e–h. Figure  5e is a typical example of the CycleGAN synthetic images like Fig. 5c. Figure 6f–h have slightly different appearances: Fig. 5f contains high-intensity artifacts surrounding each character; Fig. 5g contains wrinkle-like structures in the background; Fig. 5h is very bright for both characters and artifacts.

Two exemplary CycleGAN prediction results using real noisy THz images as the input are displayed in Fig. 6. In Fig. 6b,e, although noise is reduced, many fragments of the characters are removed or random strokes are added. Hence, only a small portion of characters are recognized. For example, in Fig. 6b only the characters “C”, “D”, “N”, “P” and “S” are correctly restored, and in Fig. 6e only the characters “D”, “G”, “R”, “N” and “S” are correctly restored. Figure 6 indicates that directly using CycleGAN for THz image denoising is insufficient.

**Table 1:** Quantitative comparison between real THz images and CycleGAN synthetic images.

Image type	Mean	Standard deviation	Total variation	Perceptual index
Real	17.834	37.001	4156848.0	n.a.
Synthetic	22.302	33.636	4024794.0	4.52

### Pix2pixGAN results

The Pix2pixGAN results of the same two exemplary THz images are displayed in Fig. 6c,f, where the Pix2pixGAN model was trained with 2000 synthetic images and tested on the real noisy THz images. For the Xuan-paper input image (Fig. 6a), its Pix2pixGAN output is entirely noiseless and all the characters in this image can be well recognized, as shown in Fig. 6c.

The result of the standard-paper input is noise-free as well in Fig. 6f. Due to the relatively high-level noise in THz images using standard paper, some parts of certain characters are missing in Fig. 6f, for example, the letter “E” and “Z”. Nevertheless, other characters like “C” and “S” are well recognized.

**Figure 7:**
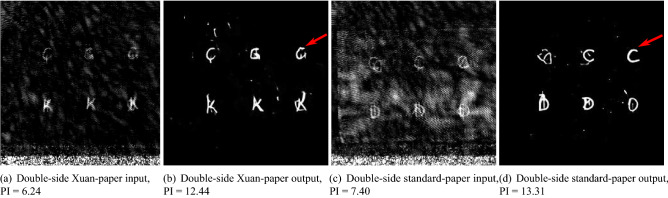
Two exemplary results of Pix2pixGAN on the real THz image written on two-side of the Xuan paper and standard paper, respectively.

Two exemplary results of Pix2pixGAN on two-sided written THz images are displayed in Fig. 7. For both Xuan and standard paper, noise (artifacts) is removed, although some residual artifacts remain in the background. Compared with characters written on the back side, those on the front side are recognized much better. Nevertheless, the interpreted letter “G” in Fig. 7b is actually either “Q” or “O” in Fig. 7a, while the letter “C” in Fig. 7d is actually a mixture of two letters in the input image Fig. 7c.

**Figure 8:**
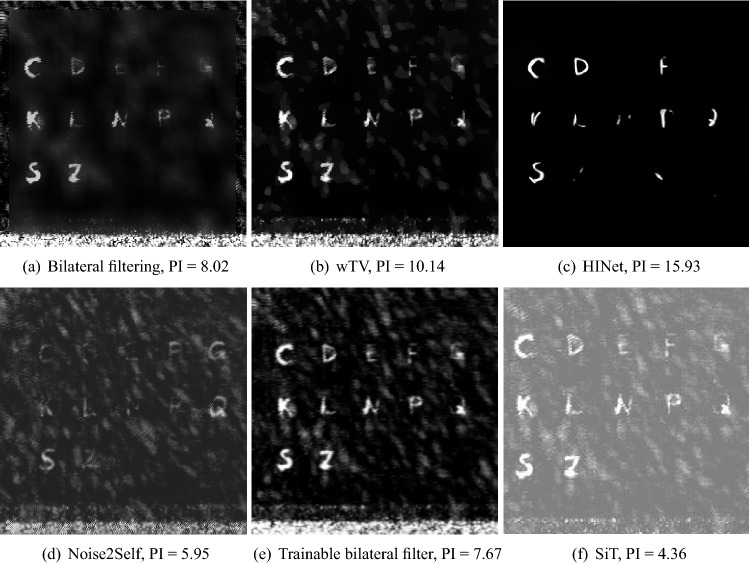
Comparison of different algorithms on the same THz image written on Xuan paper (Fig. 6a).

The results of comparison algorithms on the same THz image written on Xuan paper (Fig. 6a) are displayed in Fig. 8. Figure 8a demonstrates that a bilateral filter with hand-picked filter parameters can reduce the noise and image artifacts to some degree, but the resultant background appears blurry. In Fig. 7b, the noise and artifacts are reduced as well. However, some “shadow” artifacts remain. The HINet result in Fig. 7c has the best binarization performance, although some artifacts remain. Like bilateral filter and wTV, HINet is able to improve the image quality, but many fragments of the characters are missing. The self-supervised learning algorithms all fail to reduce noise or artifacts, as displayed in Fig. 8d–f. Therefore, they are excluded for further quantification in Table 2. The character recognition accuracy in Table 2 indicate that almost all the characters (99%) in the Pix2pixGAN results can be recognized for Xuan paper, while $\le 50\%$ characters are recognized in the results of other algorithms. For standard paper, only 61% characters are recognized in the Pix2pixGAN results. But it is still higher than the accuracies of other algorithms. The PI scores of the bilateral filtering and wTV results are smaller than the average PI (16.19) of the clean handwriting generator images, which indicates noise and artifacts remain in such images. In contrast, the PI scores of HINet are larger than 16.19, which indicates good binarization of their results. However, the missing fragments in its processed images result in sparser image features, which lead to larger PI scores. CycleGAN and Pix2pixGAN both achieve PI scores close to 16.19. However, as some characters generated by CycleGAN are meaningless, its character recognition accuracies are still low.

**Table 2:** The accuracy of character recognition and PI scores in denoised THz images with different algorithms.

Algorithm	Accuracy (Xuan), %	Accuracy (standard), %	PI (Xuan)	PI (standard)
Bilateral filtering	42	16	10.72 ± 1.17	8.49 ± 0.86
wTV	50	33	11.77 ± 0.67	9.14 ± 0.51
HINet	42	33	17.50 ± 1.18	18.80 ± 0.20
CycleGAN	41	16	14.08 ± 1.14	15.41 ± 0.75
Pix2pixGAN	99	61	16.54 ± 0.91	17.12 ± 0.50

## Discussion

CycleGAN should be able to convert clean images into noisy ones and reversely convert noisy images into clean ones in the ideal case. In our work, Fig. 5 demonstrates that CycleGAN is able to generate realistic noisy images from clean images generated by a handwriting generator. However, it is not able to generate satisfying denoised images directly from real noisy THz images as shown in Fig. 6. CycleGAN does a better job in translating clean images to noisy ones than translating noisy images to clean ones as we observed. This could be explained using the concept of entropy: getting noisy images, which have higher entropy, is easier than getting clean images, which have lower entropy. Therefore, CycleGAN is applied to generate the paired noisy image of the clean handwriting images first, and then an additional supervised-learning network trained from such paired data is applied to get the final denoised images.

Data scarcity is a common problem for deep learning applications. Generating synthetic data is commonly used nowadays for training deep learning models in various fields^57,58^, which have been demonstrated good generalizability to real data. The results in this work demonstrate that using synthetic data for training supervised deep learning models is also effective for THz image denoising. This encourages further deep learning based THz applications.

Figures 6 and 7 reveal which types of historical documents are suitable for context retrieval by THz imaging: (a) Fig. 6c demonstrates the efficacy of Pix2pixGAN in THz image denoising for one-sided Xuan paper; (b) Fig. 6f indicates that THz imaging with deep learning denoising has the potential to reveal most information written in a single-page standard paper; (c) Fig. 7 indicates that character recognition in THz images for documents with double-sided text is very challenging, regardless whether Xuan or standard paper is used.

In the real THz images, not only high-frequency noise but also image artifacts with high-intensity block-like structures exists. Conventional denoising algorithms like (trainable) bilateral filter and wTV are effective in reducing high-frequency noise. However, they are not optimal to remove structured artifacts. The HINet is also a supervised learning network using the same training data as Pix2pixGAN. It learns to binarize the real THz images from synthetic training data. However, due to its limited representation power by architecture design (design for denoising only), it is not able to restore missing fragments of the characters. The self-supervised learning networks like Noise2Self or Noise2Void consider local noise characteristics, like the J-invariant^52^. Therefore, such networks are optimized to denoise random noise based on local neighbourhoods, but not suitable for block-like structured artifacts. To develop effective self-supervised learning algorithms for such THz images, further research is required.

Some characters written on one-side-standard paper are ambiguous to recognize after Pix2pixGAN denoising, for example, the letters “E”, “F” and “G” in Fig. 6f. In our experiments, only individual characters, instead of words or sentences, are written on the pages, which increases ambiguity once any character misses fragments. Such ambiguity can potentially be reduced for words and sentences based on their surrounding context. In other words, spell correction can be performed to get meaningful words and sentences and hence reduce ambiguity. This is one potential advantage of real historical document analysis. To generate synthetic data for training, more sophisticated handwriting styles are available^44,59^. However, real historical documents contain many other challenges, for example, blurred handwriting due to aging and imaging shadow artifacts caused by paper wrinkles. Such challenges require our future exploration. Nevertheless, this work is an important step towards real nondestructive historical document analysis using THz imaging.

In this work, the CycleGAN and Pix2pixGAN models are purely data driven. Data driven deep learning models may not generalize well to out-of-distribution test data and are sensitive to noise or perturbations^49,60^. Therefore, in our CycleGAN results, some synthetic images have different appearance characteristics (e.g., Fig. 5h), which we exclude for training Pix2pixGAN. Developing physics-informed neural networks^61^, which are built based on known operators^62^ and hence can combine the advantages of both deep learning and conventional methods, for supervised learning should be investigated in our future work. Conventional THz imaging theories have the potential to develop more robust and effective neural networks for THz image enhancement. For example, the conventional mathematical modelling of THz point spread function and simulation of THz imaging systems^23^ can guide CycleGAN or a customly designed network to generate more diverse and realistic THz images^63^ for training Pix2pixGAN, which may enable Pix2pixGAN to generalize well for THz images acquired from various system settings.

## Conclusion

This work applies deep learning to denoise THz images for nondestructive historical document analysis. To overcome the data scarcity problem when training a supervised deep learning model, an unsupervised learning network, CycleGAN, is applied first to generate paired noisy images from clean synthetic images generated by a handwriting generator. Such synthetic paired data is effective to train Pix2pixGAN for THz image denoising. Our work demonstrates that the deep learning denoising performance as well as the resultant character recognition accuracy depends highly on the paper type: Context can be easily retrieved on one-side-Xuan paper after Pix2pixGAN denoising; Most context written on one-side-standard paper can still be retrieved using Pix2pixGAN; However, context written on both sides is very challenging to retrieve due to the overlap of characters. This work is an important step towards real THz-imaging-based nondestructive historical document analysis.

## Data Availability

The datasets generated and/or analyzed during the current study are not publicly available but are available from the corresponding author on reasonable request.

## References

[1] Sulaiman A, Omar K, Nasrudin MF (2019). Degraded historical document binarization: A review on issues, challenges, techniques, and future directions. J. Imaging.

[2] Padoan, R., Steemers, T., Klein, M., Aalderink, B. & De Bruin, G. Quantitative hyperspectral imaging of historical documents: Technique and applications. *Art Proc.* 25–30 (2008).

[3] Stromer D (2019). Virtual cleaning and unwrapping of non-invasively digitized soiled bamboo scrolls. Sci. Rep..

[4] Redo-Sanchez A (2016). Terahertz time-gated spectral imaging for content extraction through layered structures. Nat. Commun..

[5] Stromer D (2018). Browsing through sealed historical manuscripts by using 3-d computed tomography with low-brilliance x-ray sources. Sci. Rep..

[6] Jones C, Duffy C, Gibson A, Terras M (2020). Understanding multispectral imaging of cultural heritage: Determining best practice in MSI analysis of historical artefacts. J. Cult. Herit..

[7] Siegel PH (2002). Terahertz technology. IEEE Trans. Microwave Theory Tech..

[8] Pawar AY, Sonawane DD, Erande KB, Derle DV (2013). Terahertz technology and its applications. Drug Invent. Today.

[9] Guillet JP (2014). Review of terahertz tomography techniques. J. Infrared Millim. Terahertz Waves.

[10] Hasan M, Arezoomandan S, Condori H, Sensale-Rodriguez B (2016). Graphene terahertz devices for communications applications. Nano Commun. Netw..

[11] Kemp, M. C. *et al.* Security applications of terahertz technology. In *Terahertz for Military and Security Applications*, vol. 5070, 44–52 (SPIE, 2003).

[12] Knobloch P (2002). Medical THz imaging: An investigation of histo-pathological samples. Phys. Med. Biol..

[13] Tao YH, Fitzgerald AJ, Wallace VP (2020). Non-contact, non-destructive testing in various industrial sectors with terahertz technology. Sensors.

[14] Cosentino A (2016). Terahertz and cultural heritage science: Examination of art and archaeology. Technologies.

[15] Ullmann, I., Root, K., Schür, J., Scheuble, L. & Vossiek, M. Contactless inspection of handwritten documents with terahertz imaging. In *2021 18th European Radar Conference (EuRAD)*, 349–352 (IEEE, 2022).

[16] Labaune, J., Jackson, J., Pagès-Camagna, S., Menu, M. & Mourou, G. Terahertz investigation of Egyptian artifacts. In *35th International Conference on Infrared, Millimeter, and Terahertz Waves*, 1–3 (IEEE, 2010).

[17] Zhao J (2014). Terahertz imaging with sub-wavelength resolution by femtosecond laser filament in air. Sci. Rep..

[18] Li T, Sun Y, Shi W, Shao G, Liu J (2018). Terahertz pulse imaging: A novel denoising method by combing the ant colony algorithm with the compressive sensing. Open Phys..

[19] Ljubenović M, Zhuang L, De Beenhouwer J, Sijbers J (2020). Joint deblurring and denoising of THz time-domain images. IEEE Access.

[20] Xu L, Fan W, Liu J (2013). Suppression of the fluctuation effect in terahertz imaging using homomorphic filtering. Chin. Opt. Lett..

[21] Li, Y. & Zhao, G. Image denoising and enhancement of terahertz passive imaging. In *Eleventh International Conference on Digital Image Processing (ICDIP 2019)*, vol. 11179, 240–248 (SPIE, 2019).

[22] Cui, S.-S. & Li, Q. Research on denoising method based on guided bilateral filter for reconstructed image in terahertz holography. In *Fourth Seminar on Novel Optoelectronic Detection Technology and Application*, vol. 10697, 586–591 (SPIE, 2018).

[23] Ahi K (2017). Mathematical modeling of THz point spread function and simulation of THz imaging systems. IEEE Trans. Terahertz Sci. Technol..

[24] Ning W (2019). Resolution enhancement in terahertz imaging via deconvolution. IEEE Access.

[25] Ahi K (2019). A method and system for enhancing the resolution of terahertz imaging. Measurement.

[26] Shams M (2014). Approaching real-time terahertz imaging with photo-induced coded apertures and compressed sensing. Electron. Lett..

[27] Ren X, Bai Y, Jiang Y (2021). Hybrid sparsity model for fast terahertz imaging. Micromachines.

[28] Chan WL, Moravec ML, Baraniuk RG, Mittleman DM (2008). Terahertz imaging with compressed sensing and phase retrieval. Opt. Lett..

[29] Lu Y (2020). Reflective single-pixel terahertz imaging based on compressed sensing. IEEE Trans. Terahertz Sci. Technol..

[30] Jiang, Y. *et al.* Machine learning and application in terahertz technology: A review on achievements and future challenges. *IEEE Access* (2022).

[31] Shen Y, Yin Y, Li B, Zhao C, Li G (2021). Detection of impurities in wheat using terahertz spectral imaging and convolutional neural networks. Comput. Electron. Agric..

[32] Jiang Y (2022). Identification of unsound grains in wheat using deep learning and terahertz spectral imaging technology. Agronomy.

[33] Liu H, Vohra N, Bailey K, El-Shenawee M, Nelson AH (2022). Deep learning classification of breast cancer tissue from terahertz imaging through wavelet synchro-squeezed transformation and transfer learning. J. Infrared Millim. Terahertz Waves.

[34] Lu W (2022). Detection of heavy metals in vegetable soil based on THz spectroscopy. Comput. Electron. Agric..

[35] Wang Y, Qi F, Wang J (2021). Terahertz image super-resolution based on a complex convolutional neural network. Opt. Lett..

[36] Yang X (2022). Super-resolution reconstruction of terahertz images based on a deep-learning network with a residual channel attention mechanism. Appl. Opt..

[37] Ljubenović, M., Bazrafkan, S., Paramonov, P., Beenhouwer, J. D. & Sijbers, J. CNN-based deblurring of THz time-domain images. In *International Joint Conference on Computer Vision, Imaging and Computer Graphics*, 477–494 (Springer, 2020).

[38] Zhu Y-L, She R-B, Liu W-Q, Lu Y-F, Li G-Y (2021). Deep learning optimized terahertz single-pixel imaging. IEEE Trans. Terahertz Sci. Technol..

[39] Li, K., Stantchev, R. I. & Pickwell-MacPherson, E. Convolutional neural network based denoising method for rapid THz imaging. In *2021 46th International Conference on Infrared, Millimeter and Terahertz Waves (IRMMW-THz)*, 1–2 (IEEE).

[40] Stantchev RI, Li K, Pickwell-MacPherson E (2021). Rapid imaging of pulsed terahertz radiation with spatial light modulators and neural networks. ACS Photon..

[41] Jiang, Y. *et al.* Adaptive compressed sensing algorithm for terahertz spectral image reconstruction based on residual learning. *Spectrochim. Acta Part A Mol. Biomol. Spectrosc.*, 121586 (2022). 10.1016/j.saa.2022.121586 35853252

[42] Choi H, Kim S, Maeng I, Son J-H, Park H (2022). Improving signal-to-noise ratio of a terahertz signal using a wavenet-based neural network. Opt. Express.

[43] Jiao Q (2022). Fractional variation network for THz spectrum denoising without clean data. Fractal Fractional.

[44] Mayr, M. *et al.* Spatio-temporal handwriting imitation. In *Proc. ECCV*, 528–543 (Springer, 2020).

[45] Zhu, J.-Y., Park, T., Isola, P. & Efros, A. A. Unpaired image-to-image translation using cycle-consistent adversarial networks. In *Proc. ICCV*, 2223–2232 (2017).

[46] Isola, P., Zhu, J.-Y., Zhou, T. & Efros, A. A. Image-to-image translation with conditional adversarial networks. In *Proc. CVPR*, 1125–1134 (2017). Code: https://github.com/junyanz/pytorch-CycleGAN-and-pix2pix.

[47] Tomasi, C. & Manduchi, R. Bilateral filtering for gray and color images. In *Proc. ICCV*, 839–846 (IEEE, 1998).

[48] Wagner F (2022). Ultralow-parameter denoising: Trainable bilateral filter layers in computed tomography. Med. Phys..

[49] Wagner F (2022). Trainable joint bilateral filters for enhanced prediction stability in low-dose CT. Sci. Rep..

[50] Huang Y (2018). Scale-space anisotropic total variation for limited angle tomography. IEEE Trans. Radiat. Plasma Med. Sci..

[51] Chen, L., Lu, X., Zhang, J., Chu, X. & Chen, C. HINet: Half instance normalization network for image restoration. In *Proc. CVPR*, 182–192 (2021).

[52] Batson, J. & Royer, L. Noise2self: Blind denoising by self-supervision. In *International Conference on Machine Learning*, 524–533 (PMLR, 2019).

[53] Atito, S., Awais, M. & Kittler, J. Sit: Self-supervised vision transformer. In *Proc. ICCV*, 9650–9660 (2021).

[54] Blau, Y., Mechrez, R., Timofte, R., Michaeli, T. & Zelnik-Manor, L. The 2018 PIRM challenge on perceptual image super-resolution. In *Proceedings of the European Conference on Computer Vision (ECCV) Workshops* (2018).

[55] Mittal A, Soundararajan R, Bovik AC (2012). Making a “completely blind” image quality analyzer. IEEE Signal Process. Lett..

[56] Ma C, Yang C-Y, Yang X, Yang M-H (2017). Learning a no-reference quality metric for single-image super-resolution. Comput. Vis. Image Underst..

[57] Chen RJ, Lu MY, Chen TY, Williamson DF, Mahmood F (2021). Synthetic data in machine learning for medicine and healthcare. Nat. Biomed. Eng..

[58] Mill L (2021). Synthetic image rendering solves annotation problem in deep learning nanoparticle segmentation. Small Methods.

[59] Mattick, A., Mayr, M., Seuret, M., Maier, A. & Christlein, V. Smartpatch: Improving handwritten word imitation with patch discriminators. In *International Conference on Document Analysis and Recognition*, 268–283 (Springer, 2021).

[60] Huang, Y. *et al.* Some investigations on robustness of deep learning in limited angle tomography. In *International Conference on Medical Image Computing and Computer-Assisted Intervention*, 145–153 (Springer, 2018).

[61] Raissi M, Perdikaris P, Karniadakis GE (2019). Physics-informed neural networks: A deep learning framework for solving forward and inverse problems involving nonlinear partial differential equations. J. Comput. Phys..

[62] Maier AK (2019). Learning with known operators reduces maximum error bounds. Nat. Mach. Intell..

[63] Lyatti M (2007). Signal and noise characteristics of terahertz frequency-selective and broadband high-$$t\_ {c}$$ Josephson detectors. IEEE Trans. Appl. Supercond..

